# Recurrent Urinary Tract Infections due to Asymptomatic Colonic Diverticulitis

**DOI:** 10.1155/2012/934168

**Published:** 2012-02-19

**Authors:** Evangelos Falidas, Georgios Anyfantakis, Stavros Boutzouvis, Michail Kyriakopoulos, Stavros Mathioulakis, Konstantinos Vlachos, Constantinos Villias

**Affiliations:** ^1^1st Department of Surgery, 417 NIMTS Veterans Hospital of Athens, Athens 11521, Greece; ^2^Department of Radiology, 417 NIMTS Veterans Hospital of Athens, Athens 11521, Greece; ^3^Department of Urology, 417 NIMTS Veterans Hospital of Athens, Athens 11521, Greece

## Abstract

Colovesical fistula is a common complication of diverticulitis. Pneumaturia, fecaluria, urinary tract infections, abdominal pain, and dysuria are commonly reported. The authors report a case of colovesical fistula due to asymptomatic diverticulitis, and they emphasize the importance of deeply investigate recurrent urinary tract infection without any bowel symptoms. They also briefly review the literature.

## 1. Introduction

Colovesical fistula is a common complication of diverticular disease. Colovesical fistula appears in 2% to 22% of patients with a documented diverticulitis, while 10% to 15% of patients requiring surgical treatment for diverticulitis have fistula [1, 2]. Prior to fistula formation, diverticulosis is usually symptomatic. We report the case of a 75-year-old man presenting recurrent urinary tract infections, initially treated with antibiotics and finally attributed after an extensive investigation to colovesical fistula related to asymptomatic diverticulosis.

## 2. Case Presentation

**Figure 1:**
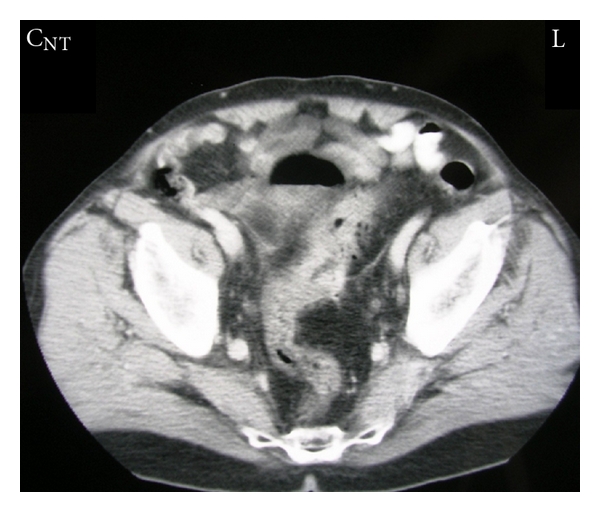
Transverse abdominal CT scan demonstrating airlevel into the urinary bladder closely in contact with a thickened segment of the sigmoid colon and colon diverticula.

**Figure 2:**
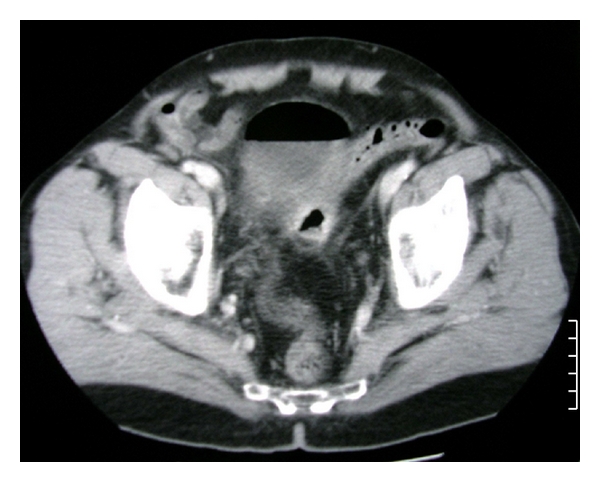
Transverse abdominal CT scan demonstrating airlevel into the urinary bladder closely in contact with a thickened segment of the sigmoid colon and colon diverticula.

A 75-year-old man came to the emergency department complaining of high fever of 4-day duration and urgency in urination. He mentioned recurrent and multiple (five) episodes of urinary tract infections over the last 3 months attributed to a recent (12 months ago) transurethral prostatectomy. These episodes were resolved with oral administration of various antibiotics. Escherichia coli was constantly found in urine cultures. However, no history of abdominal pain, fever, particular changes of defecation habits, pneumaturia, or fecaluria was referred. No diverticulosis or diverticulitis was identified in prior medical examinations. Repeated urinary tract ultrasound controls were undertaken in order to exclude prostate, urinary blabber, uretere, or kidneys abnormalities or lithiasis without particular abnormal findings. On admission, leukocytosis (17000 mm^3^) was the unique abnormal laboratory finding. Pyuria and hematuria were found in urinalysis. Upon palpation, no abdominal pain or positive Giordano maneuver were observed. Urine and blood cultures were taken. He received intravenously fluids and empirical wide spectrum antibiotic treatment (ciprofloxacin). Temperature was completely normalized 2 days later. Urine cultures revealed Escherichia coli and Pseudomonas aeruginosa (colony-forming units (CFU) >10^6^/mL of urine) sensitive to ciprofloxacin. No atypical antibiotic sensitivities or resistances were observed according to the microbiologic data of our hospital. Cystoscopy demonstrated redness of the lateral wall of the bladder without any fistula identified. Cystography also did not demonstrate any fistula tract. However, abdominal computed tomography scan (CT) revealed the presence of free air within the urinary bladder in contact with perisigmoid thickening and colon diverticula (Figures 1 and 2). In addition, colonoscopy was performed in order to exclude colonic neoplasm. No neoplasm was identified; however, diffuse diverticula of the sigmoid colon with thickness, edema, and inflammation into the lumen were observed. No evident fistula tract was revealed.

Elective surgery was performed. Upon laparotomy, pericolonic thickening was noted at the level of the inferior segment of the sigmoid colon strongly adherent to urinary bladder lateral wall. Blunt dissection was performed and a sigmoidectomy followed by an end-to-end anastomosis. The fistula tract was identified and a tiemann catheter was inserted. Urinary bladder was opened extraperitoneally and the point of the colovesical fistula was identified. The fistula was resected around the catheter. No postoperative complications occurred. Foley catheter was removed eight days later. He was discharged ten days after the initial observation.

## 3. Discussion

Colovesical fistula is a common complication of large bowel diverticulosis. Incidence ranges from 2 to 22% of patients with a known large bowel diverticulosis [1]. In addition, 10–15% of patients requiring surgical intervention for colonic diverticulosis have a symptomatic colovesical communication [2]. Up to 75% of colovesical fistulas are associated with colon diverticulitis [3]. It usually concerns patients older than fifty years and mainly men. Male predominance could be attributed to a relative protective effect of uterus on urinary bladder in women [4]. Crohn's disease, colon or bladder cancer, radiotherapy, and iatrogenic injuries may also favor colovesical fistula formation [3]. Pneumaturia and fecaluria are the most common symptoms, while recurrent urinary tract infections, abdominal pain, and dysuria are also frequently referred. Patients may have colovesical fistula and may not have significant abdominal symptoms. Orchitis due to colovesical fistula have also been reported [5]. In most cases, the fecal content spread into the urinary bladder and rarely vice versa. In our case, the first episode of urinary tract infection was observed only 3 months ago without previous signs or symptoms of bowel dysfunction or diverticulitis.

Clinical history and patient's complaints of pneumaturia and fecaluria are often enough to raise the suspicion of a colovesical fistula. Routine urine tests usually reveal colonic microorganisms as well as cellulose fibers and/or orally administered contrast essences (charcoal, methylene-blue, or barium). CT is diagnostic, in 90–100% of cases, revealing air or contrast material into the urinary bladder. In addition, CT scan reveals associated abnormalities such as neoplasms or inflammatory processes [3]. Cystoscopy or barium enema seems to be less specific to the diagnosis of the disease (38–48%) [1, 3]. Other diagnostic test such as pyelography, cystogram, and ultrasonography present a relative diagnostic value [3]. Colonoscopy is important in excluding concomitant abnormalities, although it frequently does not visualize the fistula [1, 3]. In our case cystoscopy as well as cystogram did not identify fistula. CT confirmed diagnosis, while colonoscopy excluded neoplasms and revealed diverticulitis without any fistula tract identified. 

Surgical treatment with colonic resection and immediate anastomosis (one stage procedure) is the recommended treatment, while staged repairs are indicated in cases of associated pelvic abscesses, malignancies, or radiation alterations. Primary closure of the cystic defect, flap of the omentum, resection, and closure are acceptable surgical options [4, 6]. Colostomy is a palliative approach that limits the fecaluria and improves urinary infection; however, urine passage into the distal colonic segment may be more disabling than the fistula itself [7]. Laparoscopic excision has been successfully achieved, although no additional benefits have been proved in terms of morbidity and long-term results. In addition, conversion in laparotomy is common [8]. Conservative treatment with intermittent administration of wide-spectrum antibiotic use could be an alternative in patients who do not wish to be operated or present a high surgical risk because of age or comorbidities [9]. In our case, five severe episodes of urinary tract infection in three months and the absence of significant comorbidities made us opt for the one-stage procedure. 

## 4. Conclusion

Colovesical fistula is common complication of colon diverticulitis or colon cancer. Multiple and recurrent urinary tract infections with no other underlying pathologies should always raise suspicion of colovesical fistula. Asymptomatic diverticulitis may be the cause of colovesical fistula.
